# Inflammatory Biomarkers Interleukin 1 Beta (IL-1β) and Tumour Necrosis Factor Alpha (TNF-α) Are Differentially Elevated in Tobacco Smoke Associated COPD and Biomass Smoke Associated COPD

**DOI:** 10.3390/toxics9040072

**Published:** 2021-04-01

**Authors:** Bellipady Shyam Prasad Shetty, Sindaghatta Krishnarao Chaya, Sravan Kumar V, Maheswarappa Mahendra, Biligere Siddaiah Jayaraj, Komarla Sundararaja Lokesh, Koustav Ganguly, Padukudru Anand Mahesh

**Affiliations:** 1 Department of Cardiothoracic and Vascular Surgery, JSS Medical College and Hospital, JSS Academy of Higher Education & Research, Mysuru 570015, India; nivishyam@gmail.com; 2 Department of Respiratory Medicine, JSS Medical College and Hospital, JSS Academy of Higher Education & Research, Mysuru 570015, India; chaya.sindaghatta@gmail.com (S.K.C.); drjayarajbs@yahoo.com (B.S.J.); kslokesh@gmail.com (K.S.L.); 3 Department of Respiratory Medicine, Malla Reddy Medical College for Women, Hyderabad 500055, India; drsravanvemulapalli@gmail.com; 4 Department of Respiratory Medicine, Shimoga Institute of Medical Sciences, Shimoga, Karnataka 577204, India; mahesims@gmail.com; 5 Unit of Integrative Toxicology, Institute of Environmental Medicine (IMM), Karolinska Institutet, 17177 Stockholm, Sweden; koustav.ganguly@ki.se; 6 Special Interest Group-Environment and Respiratory Diseases, JSS Academy of Higher Education & Research, Mysuru 570015, India

**Keywords:** COPD, interleukin 1 β, tumour necrosis factor α, tobacco smoke, biomass, FEV1

## Abstract

Chronic obstructive pulmonary disease (COPD), the leading cause of mortality and morbidity worldwide, is characterized by abnormal activation of inflammatory cells. The increased pro-inflammatory cytokines, such as tumour necrosis factor alpha (TNF-α) and interleukin 1 beta (IL-1β), further amplify the inflammation. We evaluated the dose response relationship of IL-1β and TNF-α levels and severity of airflow limitation, and differential responses in IL-1β and TNF-α between biomass COPD (BMS-COPD) and tobacco smoke COPD (TS-COPD) using a case control design in 160 subjects. Patients with COPD had higher serum levels of both IL-1β and TNF-α compared to healthy controls. A large difference in TNF-α was observed between TS-COPD and BMS-COPD, where TS-COPD patients had much higher levels. Serum IL-1β levels were higher in BMS-COPD. Levels of IL-1β correlated better with severity of airflow limitation than TNF-α levels. Both TNF-α and IL-1β levels had a negative linear relationship with Forced Expiratory Volume in 1st second (FEV1) and six-minute walk distance. The correlations were stronger with FEV1 than six-minute walk distance. The correlations of TNF-α and IL-1β with St George Respiratory Questionnaire (SGRQ) scores and body mass index (BMI) were not significant. In conclusion, the levels of pro-inflammatory cytokines TNF-α and IL-1β are differently elevated in TS-COPD and BMS-COPD, respectively.

## 1. Introduction

Chronic obstructive pulmonary disease (COPD) is one of the leading causes of morbidity and mortality worldwide and is the 4th leading cause of death in adults aged above 50 years [1]. COPD was the second leading cause of disease burden in India, contributing 8.7% of the total deaths and 4.8% of the total disability adjusted life years (DALYs) [2]. Prevalence of COPD in adults aged ≥40 years is ∼9–10% worldwide, and in India, it is ranged between 6.5 and 7.68% [3,4,5]. It is characterized by chronic inflammation with progressive obstruction of expiratory flow affecting large and peripheral airways, together with fibrosis and tissue damage [6]. COPD is also associated with systemic responses such as oxidative stress, activation of circulating inflammatory cells, and increased levels of cytokines [7,8]. Systemic manifestations of COPD are expressed as metabolic abnormalities, weight loss, muscle weakness and wasting, cardiovascular disease, depression, osteoporosis, cancer, and anaemia [9]. The role of various cytokines needs to be understood and there is a potential for development of biologicals or small molecules targeting crucial cytokines involved in chronic obstructive pulmonary disease.

Of the elevated pro-inflammatory cytokines in COPD, interleukin 1 beta (IL-1β) and tumour necrosis factor alpha (TNF-α) are considered important. Although TNF-α has been linked with physiologic proliferation and differentiation of B cells under steady-state conditions, elevated levels have been linked with a wide variety of diseases including COPD and amplify inflammation through the activation of cells’ nuclear factor kappa-light-chain-enhancer of activated B (NF-kB), activator protein 1 (AP-1), and other transcription factors [10]. Administration of TNF-α induces pathologic features typical of COPD in animal models, with similar inflammatory cell infiltrate in the lungs, which leads to emphysema [11,12]. Interleukin-1 beta (IL-1β) is a member of the interleukin-1 family that has a broad spectrum of both beneficial and harmful biological actions. IL-1β is one of the important cytokines synthesized by monocytes’ phagocytes, involved in initiation and perpetuation of inflammation [13]. IL-1β has similar actions to TNF-α and is a potent activator of alveolar macrophages from COPD patients causing disruption of alveolar septa and fibrosis in airway walls [14].

It is hypothesized, in COPD, that the inflammatory process originates in the airways and lung parenchyma, which spills over into the systemic circulation, resulting in inflammation. However, there have been inconsistent results in the studies that looked into the correlation between lung inflammation and systemic inflammation as well as the corresponding cytokine balance in COPD. It is also considered that the systemic inflammation in COPD might be primarily driven by tobacco smoking and biomass fuel smoke exposure. Several pro-inflammatory cytokines, T-cell cytokines, chemokines, growth factors, and anti-inflammatory cytokines have been implicated in COPD pathogenesis. For example, IL-1β and TNF-α enhance COPD by increasing inflammation [15]. The objective of this study was to evaluate whether there is a dose response relationship of IL-1β and TNF-α levels with COPD airflow limitation staging according to the Global Initiative for Chronic Obstructive Lung Disease (GOLD) criteria and whether there are differential responses in these biomarkers between biomass smoke COPD (BMS-COPD) and tobacco smoke COPD (TS-COPD).

## 2. Materials and Methods

Study design and study population: A prospective case control study was carried out in patients attending a tertiary care University teaching hospital in South India between July 2020 and December 2020. Ethical clearance for the study was obtained from the institution ethics committee of JSS Medical College (Letter No. JSSMC/IEC/090620/17 NCT/2020-21 dated 12 June 2020). All subjects were enrolled after an informed consent process. Cases were subjects with COPD (*n* = 80) and controls were non-COPD subjects (*n* = 80) fulfilling the inclusion and exclusion criteria. Subjects with COPD and no other comorbidity, who were current smokers (an adult who is currently smoking) with a smoking history of >10 pack years and COPD subjects with biomass smoke index ≥40 were included as cases in the TS-COPD and BMS-COPD groups, respectively. Pack years of smoking was calculated as “average number of cigarettes smoked per day multiplied by number of years of smoking divided by 20” [16]. Biomass smoke index was calculated by multiplication of average hours of exposure to biomass smoke per day multiplied by number of years of exposure [17,18]. COPD was diagnosed and airflow limitation was categorized according to GOLD criteria “post bronchodilator forced expiratory volume in 1 s/forced vital capacity (FEV1/FVC) ratio of <0.7” and staged as mild, moderate, severe, and very severe airflow limitation [19]. Subjects with COPD, who did not have an acute exacerbation in the last three months, who had not received systemic steroids during the last 1 month, and did not have any acute infection in the last three months, were included in the study.

Healthy controls were selected from the Burden of Obstructive Lung Disease (BOLD) cohort from the general population. Subjects without COPD and without any comorbidity, who were non-smokers (an adult who has never smoked in his lifetime) or smokers with <10 pack years of smoking and biomass smoke index <40 were taken as controls.

### 2.1. Data Collection

A pretested validated form, which included data on demographic details, smoking pack years, relevant present, past, personal history, and clinical measurements such as body mass index (BMI), pulse rate, blood pressure, respiratory rate, and oxygen saturation, was recorded. St. George respiratory questionnaire (SGRQ), six-minute walk test, chest X-ray, and 2D echocardiography were performed for each patient. Spirometry (Easy one PC, NDD, Medizintechnik AG, Zurich, Switzerland) was done by trained staff as per American Thoracic Society (ATS) guidelines [20]. Pre and post bronchodilator tests were done for all patients. Post bronchodilator test was done after 15 min after using 400 mcg of salbutamol. The predicted values for FEV1 and FVC were obtained using corrected Knudson’s predicted equation for Asian population (Knudson 83 × 0.87). A record of FEV1/FVC ratio (post bronchodilator) less than 0.70 was categorized as obstructive pattern and confirmed COPD according to GOLD criteria [19]. The severity of airflow limitation was classified as mild (FEV1 >80% of predicted), moderate (FEV1 50–80% of predicted), severe (FEV1 30–50% of predicted), and very severe (FEV1 <30% of predicted) as per GOLD criteria. Six minute walk test (6 MWT) was done by a trained physiotherapist in a 100 feet hallway after explaining the objective of the test according to American thoracic society guidelines [21]. Multidimensional body mass index (BMI (B), airflow obstruction (O), dyspnea (D), and exercise tolerance (E) (BODE) index was calculated [22]. 

Vitals were recorded both before and after the six minute walk test, which included pulse rate, blood pressure, respiratory rate and oxygen saturation. Test was immediately stopped if patient developed any of the following: chest pain, intolerable dyspnoea, leg cramps, staggering, sweating or pale appearance. Distance walked was recorded in a worksheet and percentage of predicted distance was calculated. Echocardiography was performed by a technician trained in echocardiography, standard two dimensional views to determine left ventricular ejection fraction (LVEF), right ventricular end diastolic diameter, left atrial and ventricular dimension, right ventricular systolic pressure (RVSP) measured by tricuspid regurgitation jet, and valve dimensions were recorded and reviewed by a cardiologist. Blood samples were obtained from the antecubital vein. The blood was centrifuged immediately at 4 °C and stored at −80 °C. Plasma levels of IL-1β and TNF-α were measured by the enzyme-linked immunosorbent assay method using Diaclone ELISA kits.

### 2.2. Statistical Analysis

Descriptive data is presented as frequencies (percentages) for discrete variables and as mean (standard deviation) for continuous variables. For comparisons between two groups, Mann–Whitney *U* test was used or, when appropriate, the two-sample t-test. Χ^2^-test was used to evaluate categorical factors. Linear regression was done to evaluate the association between cytokine levels and subgroups with adjusting for BMI. All statistical tests were 2-tailed, and factors were considered statistically significant at *p* < 0.05. IBM SPSS version 22 and CDC Epi Info version 7 was used for analysis. 

## 3. Results

**Figure 1:**
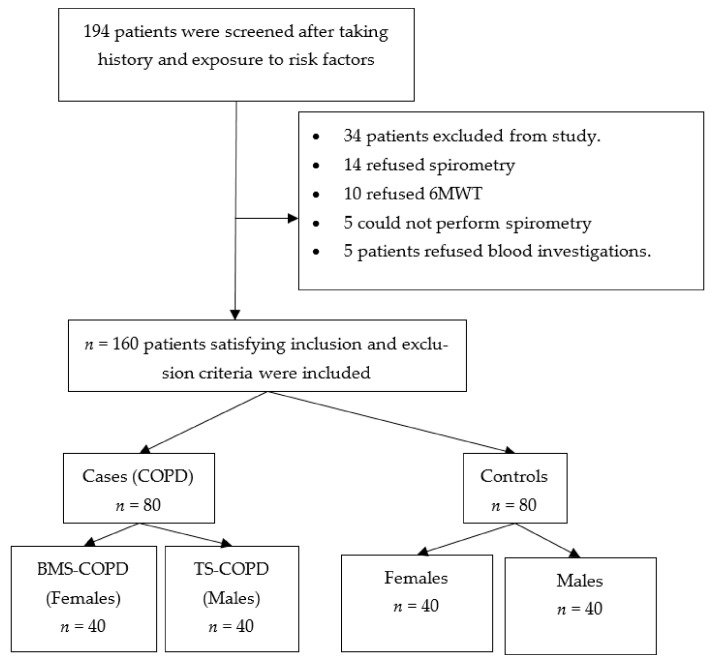
Flowchart of screening and inclusion of cohort. 6 MWT: six minute walk test, BMS: Biomass Smoke, COPD: Chronic obstructive pulmonary disease, TS: Tobacco smoke.

During the study period, a total of 194 subjects were screened for inclusion and exclusion criteria and 160 subjects were included in the study. The details of screening and the reasons for exclusion are presented in Figure 1. Among the cohort, the number of female subjects was 80 and the number of male subjects, 80. Mean age of the study population was 58.20 ± 9.75. Eighty subjects with COPD (40 TS-COPD and 40 BMS-COPD) were included as cases. All the male cases were heavy smokers with the mean burden of tobacco smoking of 35.55 pack years. The clinical and demographic characteristics of the study subjects are shown in the Table 1. Significant differences were observed between cases and controls for all the variables except age. The female cases had significant exposure to biomass fuel smoke with the mean biomass smoke exposure index of 78.9. None of the COPD subjects were on regular inhaled medications as suggested in GOLD document. They used oral medications intermittently when they had acute symptoms. Eighty subjects without COPD were controls. Among the male controls, most were non-smokers, except for 2 subjects who were ex-smokers and had quit smoking more than 10 years before and had limited burden of tobacco smoke exposure of 0.28 pack years. Cases had 55% lower six min walk distance compared to controls, the mean distance was 305.53 metres and 550.53 metres, respectively. The differences between the subgroups TS-COPD and BMS-COPD are presented in Table 2. There was no significant difference in age between BMS-COPD and female controls (*p* value 0.5) as well as TS-COPD and male controls (*p* value 0.1). There was no significant difference in BMI between BMS-COPD and female controls (*p* value 0.07). There was a significant difference in BMI between TS-COPD and male controls (*p* value 0.02). Compared to BMS-COPD, subjects with TS-COPD had significantly lower BMI and a higher BODE index.

**Figure 2:**
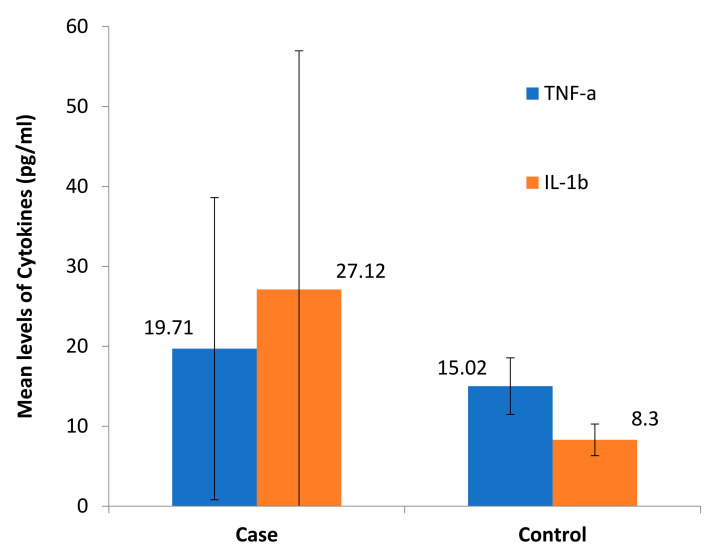
Comparison of tumour necrosis factor alpha (TNFα) (pg/mL) and interleukin 1 beta (IL-1β) (pg/mL) levels in COPD cases and controls. The difference in the IL-1β levels between cases and controls was significant (*p* value 0.0000) and the difference in the TNF-α levels was not significant (*p* value: 0.92).

**Figure 3:**
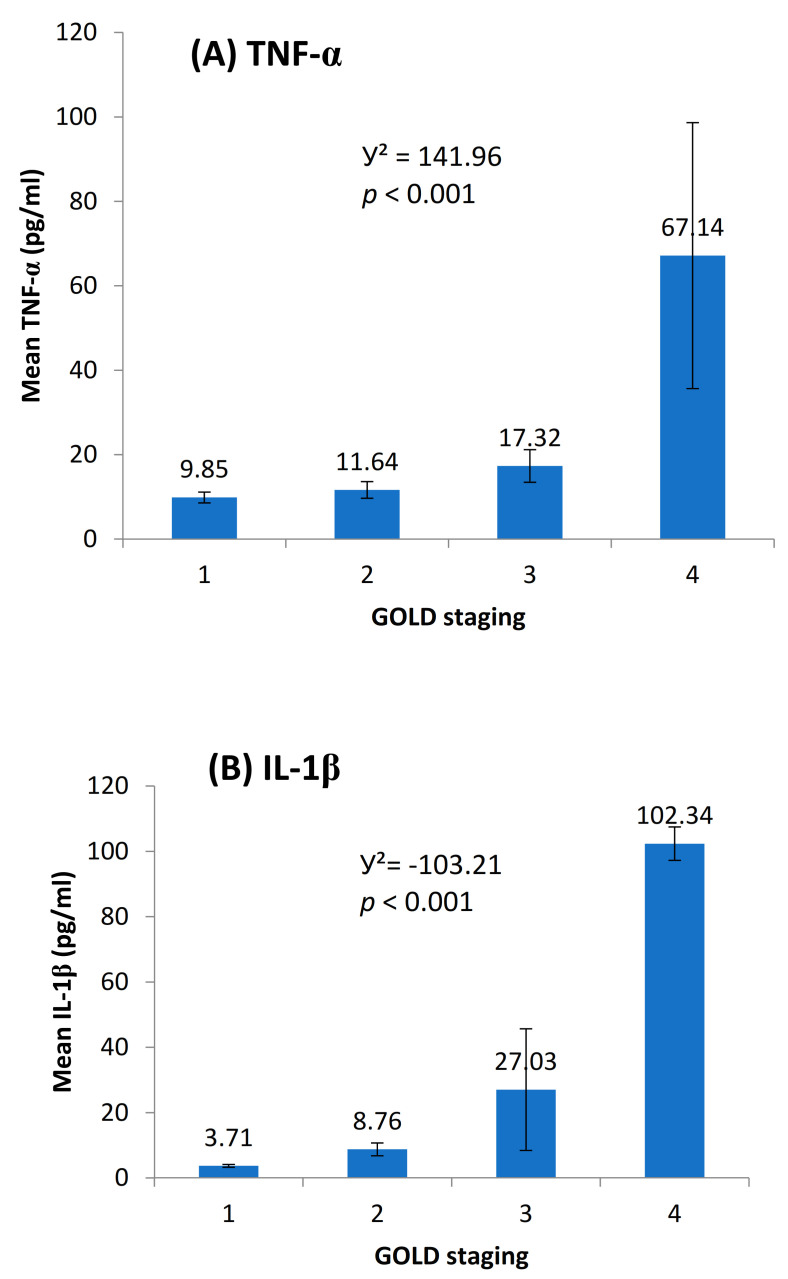
Dose response relationship between (**A**) tumour necrosis factor alpha (TNF-α) (pg/mL), (**B**) interleukin 1 beta (IL-1β) (pg/mL), and global initiative for Chronic Obstructive Lung Disease GOLD staging among COPD cases. The *p* values for the difference in mean levels of TNF-α and IL-1β between GOLD staging are: Stage 1 vs. Stage 2: TNF-α 0.81, IL-1β 0.67; Stage 1 vs. Stage 3: TNF-α 0.31, IL-1β 0.032; Stage 1 vs. Stage 4: TNF-α 0.000, IL-1β 0.000; Stage 2 vs. Stage 3: TNF-α 0.20, IL-1β 0.000; Stage 2 vs. Stage 4: TNF-α 0.000, IL-1β 0.000; Stage 3 vs. Stage 4: TNF-α 0.000, IL-1β 0.000.

**Figure 4:**
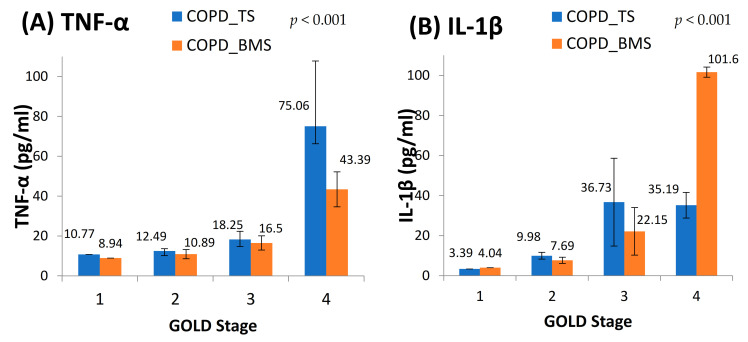
Dose response relationship between (**A**) TNF-α (pg/mL), (**B**) IL-1β (pg/mL), and GOLD staging among smoking COPD and biomass COPD cases. The *p* values for the difference between the mean levels of TNF-a among the TS-COPD and BMS-COPD were 0.32 for Stage 1, 0.017 for Stage 2, 0.176 for Stage 3, and 0.202 for Stage 4. The *p* values for the difference between the mean levels of IL-1β among the TS-COPD and BMS-COPD were 0.32 for Stage 1, 0.0003 for Stage 2, 0.016 for Stage 3, and 0.0455 for Stage 4.

**Figure 5:**
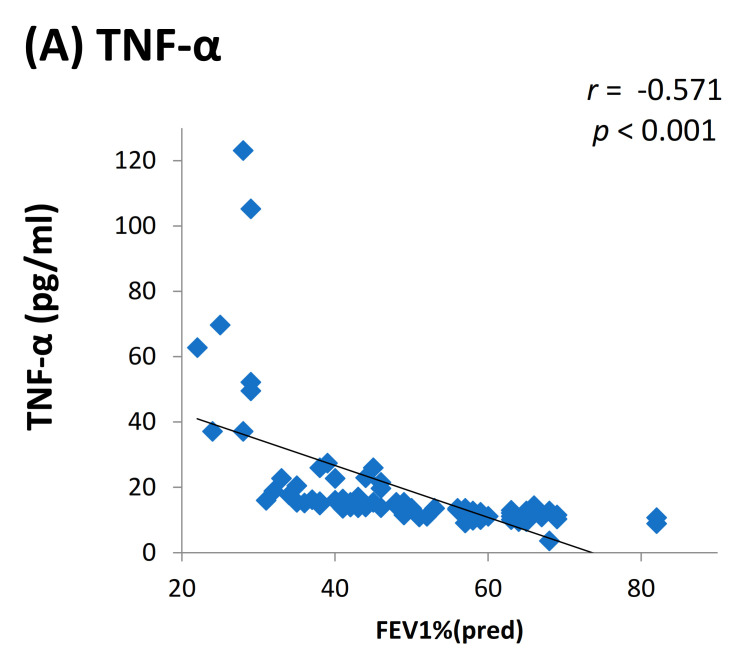

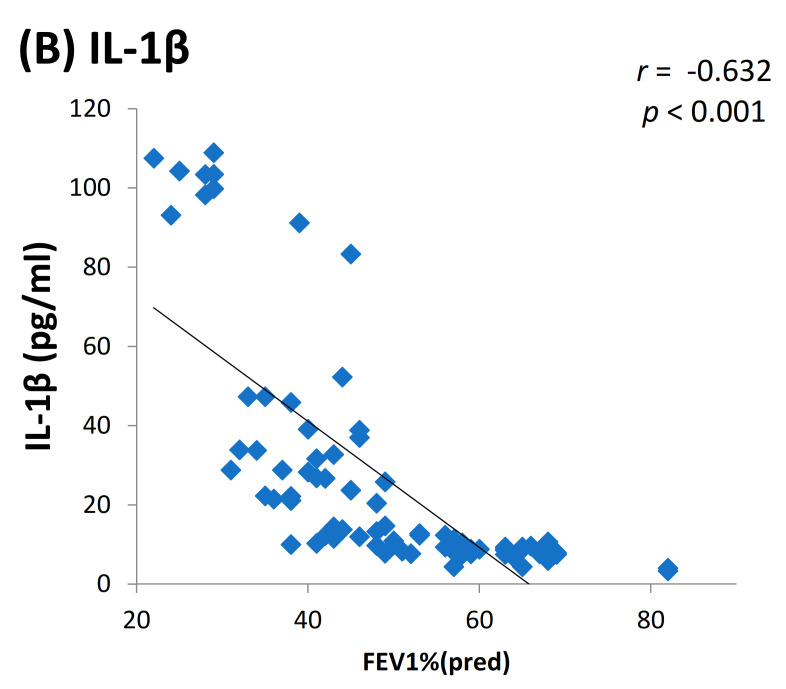
Correlation between levels of (**A**) TNF-α (pg/mL), (**B**) IL-1β (pg/mL), and Forced expiratory volume is 1st second FEV1%(pred) among the cases.

**Figure 6:**
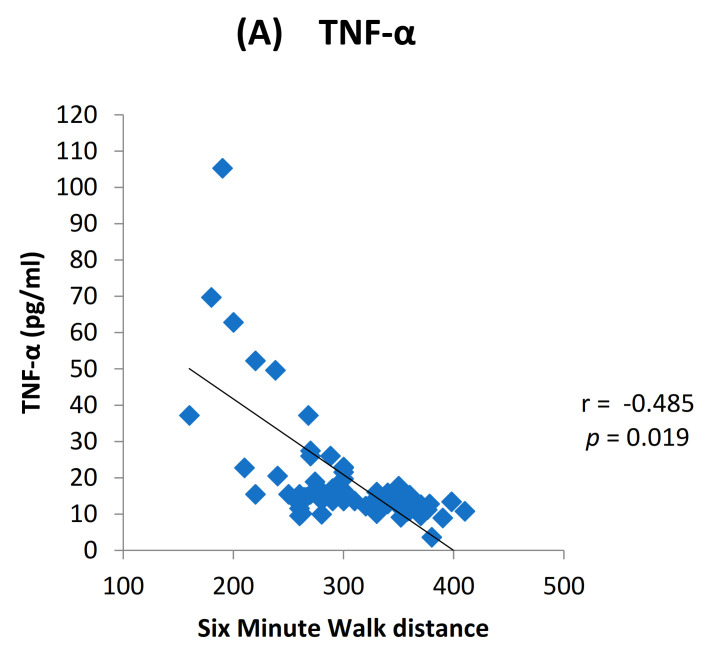

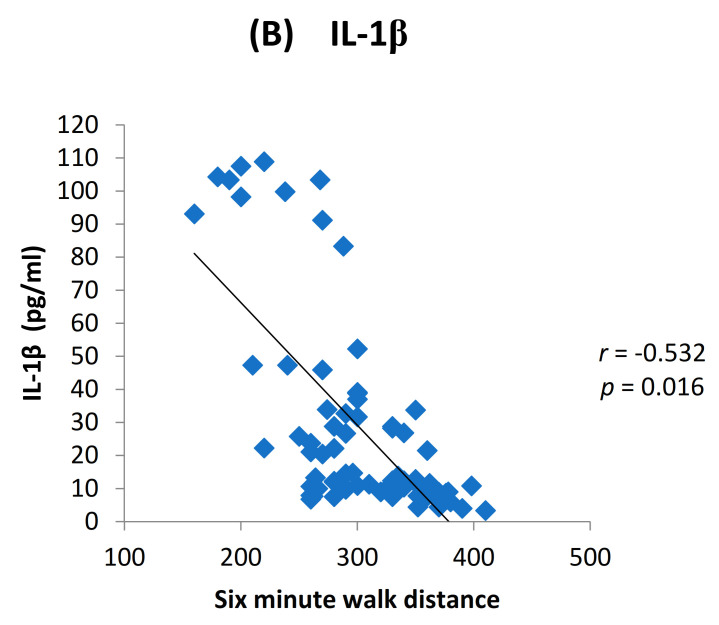
Correlation between Six min walk test and levels of (**A**) TNF-α (pg/mL) and (**B**) IL-1β (pg/mL) among the cases.

**Figure 7:**
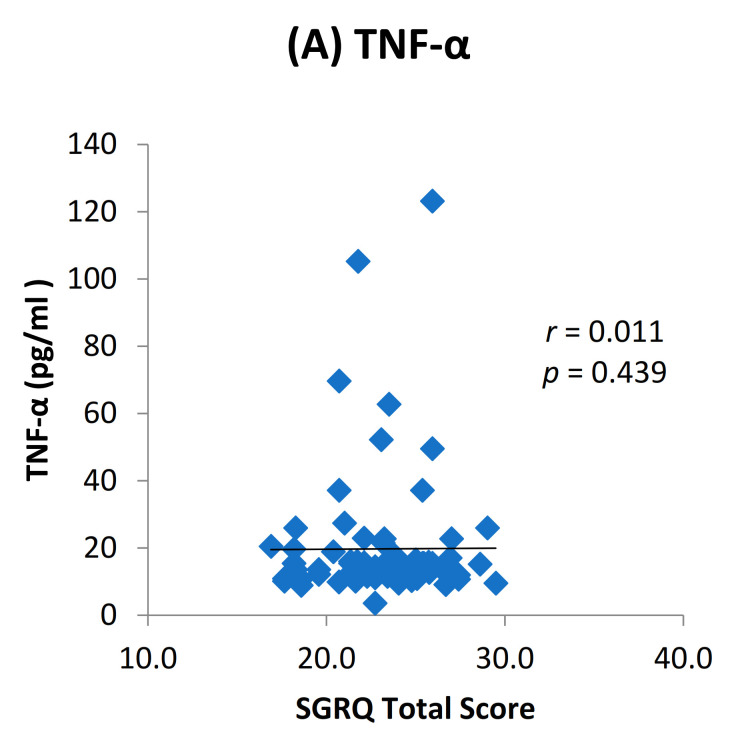

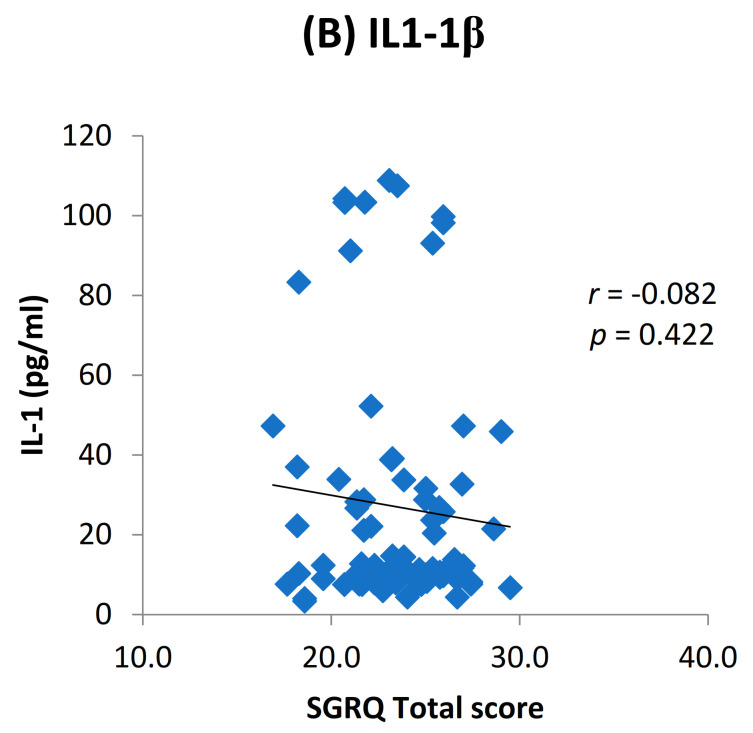
Correlation between St George Respiratory Questionanire (SGRQ) total score and levels of (**A**) TNF-α (pg/mL) and (**B**) IL-1β (pg/mL) among the cases.

**Figure 8:**
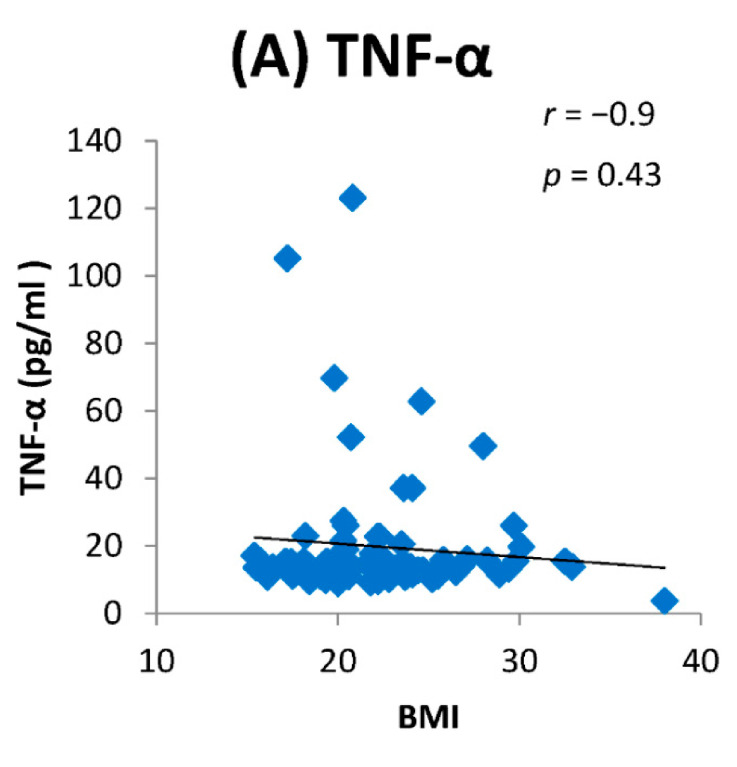

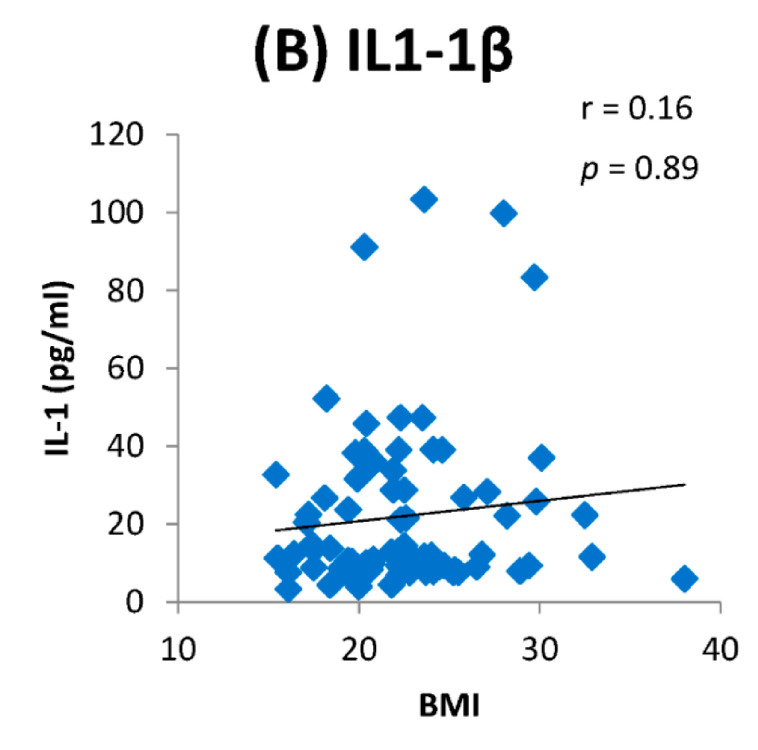
Correlation between SGRQ total score and levels of (**A**) TNF-α (pg/mL) and (**B**) IL-1β (pg/mL) among the cases. BMI: Body mass index.

We found elevated levels of IL-1β and TNF-α in cases compared to controls which were statistically significant (Figure 2). Among the cases, we found a positive dose response relationship between the levels of TNF-α and IL-1β with later stages of airflow limitation (GOLD stage 2 to 4) and not with early stage (Figure 3). On subgroup analysis of TS-COPD and BMS-COPD, a differential response was observed for TNF-α and IL-1β. TNF-α was higher in TS-COPD and IL-1β was higher in BMS-COPD (Figure 4). On correlation analysis, there was a significant negative linear relationship between TNF-α and IL-1β with FEV1 (Figure 5) as well as six-minute walk distance (Figure 6). We found no significant linear relationship between TNF-α and IL-1β with SGRQ scores (Figure 7) and BMI (Figure 8).

On linear regression analysis, TNF-α levels were significantly associated with TS-COPD but not BMS-COPD. IL-1β levels had a stronger association with both TS-COPD and BMS-COPD. There was a significant difference between TS-COPD and BMS-COPD. On adding BMI to the regression analysis, the confounding effect observed was minimal. The coefficients and *p* values are shown in Table 3.

## 4. Discussion

COPD is one the major causes of morbidity and mortality worldwide. Even though the current GOLD document does not mention inflammation as an essential component of the definition of COPD, many studies have shown the presence of pulmonary and systemic inflammation [23]. The principal abnormalities in the airways of COPD patients are the presence of a persistent inflammatory response as well as a structural re-modelling that thicken the airway wall and the destruction of alveoli leading to emphysema [24]. Noxious particles cause epithelial damage which causes activation of innate and adaptive immune cells which are responsible for the release of proteases, cytokines/chemokines, and mediators, which lead to inflammation, re-modelling, and pulmonary damage [7]. COPD is a systemic inflammatory disease and is characterized by the abnormal activation of inflammatory cells and the abnormal increase of circulating cytokines [25]. In our previous study, increased concentrations of IL-4 and TNF-α have been observed in TS COPD subjects. Both IL-4 and TNF-α showed an inverse correlation with lung function parameters FEV1/FVC and FEV1 [26].

Pro-inflammatory cytokines, such as TNF-α, IL-1, and IL-6, are increased in COPD, and appear to amplify inflammation, through the activation of the transcription factor, nuclear factor (NF)-kB, thereby leading to the increased expression of multiple inflammatory genes [15]. We observed an increase in the serum levels of both IL-1β and TNF-α in patients with COPD as compared to healthy controls. IL-1β, a pro-inflammatory cytokine synthesized by macrophages, is increased in airways during exacerbation of smoking related COPD [8] and correlates significantly with other inflammatory mediators and cells when COPD is stable [27]. Animal studies on mice have shown that IL-1β production was significantly up-regulated in lung homogenates after tobacco smoke exposure [28]. Similar results were translated in human studies where researchers found positive dose response relation between elevated IL-1β and TNF-α levels and cigarette smoking [29]. IL-1 β expression in neutrophils of COPD patients correlated with disease severity as measured by FEV1 [27]. In addition, sputum IL-1β was shown to be a potential biomarker for bacteria associated exacerbations of COPD [30]. IL-1β plays a central role in the regulation of immune responses and inflammatory processes, including promotion of the movement of inflammatory cells from the blood to inflamed tissues, regulation of the extracellular matrix, induction of the expression of a variety of inflammatory mediators such as and TNF-α, and promotion of the differentiation of inflammatory cells [31]. IL-1β and TNF-α stimulate macrophage and bronchial epithelial cells to produce matrix metalloprotein-9 and extracellular matrix protein-tenascin, respectively, which are involved in the pathogenesis of emphysema [8]. Earlier studies have observed higher levels of TNF-α [32,33,34,35,36] and IL-1β [37] in serum, bronchoalvolar lavage (BAL), and sputum of COPD subjects as compared to normal subjects. A dose response with increasing levels of serum TNF-α and IL-1β with increasing severity, as seen according to the GOLD staging of airflow obstruction, has been shown [38,39,40].

We observed large differences in TNF-α between smoke (TS) COPD and biomass smoke (BMS) COPD, with much higher levels of serum TNF-α in TS COPD. Studies comparing between TS COPD and BMS COPD using parametric response mapping (PRM) have observed that, while airways are predominantly involved in BMS COPD, emphysema is much higher in TS COPD [41]. Animal studies have confirmed that administration of TNF-α induces emphysematous changes in the lungs. TNF-α is a pro-inflammatory cytokine that is found to be elevated in COPD subjects [42]. Experimental animal models show that TNF-α over expression induces the pathological changes similar to emphysema and pulmonary fibrosis [43]. For example, in mice airspace enlargement, loss of small airspaces, increased collagen, thickened pleural septa, and increased chest and lung cavity volume are some of the changes mediated by TNF-α over-expression. This could explain the higher levels of TNF-α in TS-COPD as compared to BMS-COPD. We also observed large differences between serum levels of IL-1β between BMS-COPD and TS-COPD, with higher levels noted in BMS COPD along with a dose response relationship of serum levels of IL-1β to GOLD staging of COPD. IL-1β is shown to be associated with neutrophilic inflammation of the airways. Sputum examination of biomass smoke exposed women show greater neutrophilic inflammation as compared to clean fuel users with evidence of neutrophilic activation [44]; circulating neutrophils show an increased surface expression of CD16 (F_C_γ receptor III), β_2_ Mac-1 integrin (CD11b/CD18) and CD35 (complement receptor-1), double the levels of neutrophil chemoattractant cytokine IL-8, higher myeloperoxidase activity from circulating neutrophils, and that inducible nitric oxide synthase (iNOS) was expressed more in neutrophils from airways of biomass exposed women, who also had double the concentration of serum nitric oxide.

BMS-COPD and TS-COPD are quite distinct from one another [41,45,46,47,48]. TS-COPD has much greater emphysema which may be related to increased TNF-α level. BMS-COPD exhibits greater airway involvement with greater bronchial thickening, airway fibrosis, anthracosis, and bronchiectasis as compared to TS-COPD. Macrophages from biomass exposed individuals show significant vacuolation, which is not a usual finding seen in TS-COPD. Vascular involvement is greater in BMS-COPD; greater arteriolar wall thickening, and intense perivascular inflammatory cell (neutrophil and macrophage) infiltrates with activation of the Jun Kinase pathway result in greater incidence and severity of pulmonary hypertension and cor-pulmonale. We observed a differential elevation of TNF-α (greater in TS COPD) and IL-1β (greater in BMS COPD) with a significant dose response relationship with severity of COPD, with a large increase in late stage COPD (GOLD IV). Elevated TNF-α levels in an earlier study has been associated with body weight losing COPD [49]. In our study, we observed that elevated TNF-α levels were not significantly associated with lower BMI in both TS-COPD and BMS-COPD.

### Strengths and Weaknesses

This is one among the few studies that has compared two important inflammatory cytokines, TNF-α and IL-1β, in BMS COPD and TS COPD in India; we studied association with objective measurements such as spirometry and 6 min walk distance and subjective measurements such as quality of life. The study also evaluated whether TNF-α or IL-1β could be prognostic markers and evaluated whether increasing severity of COPD is associated with increasing levels of these novel biomarkers, which could be a useful adjunct to spirometry or in subjects unable to perform spirometry, this could be a useful surrogate. Nonetheless, we were able to observe the important association observed with increasing TNF-α and IL-1β and increasing severity of COPD, which needs to be studied in longitudinal cohorts to understand the magnitude of changes that identify clinical relevance of disease progression. As a tertiary care hospital, we were able to accurately diagnose COPD; our study subject characteristics may not be a representative of primary and secondary care population with COPD. Although this could have affected the results, consecutive subjects with COPD were screened for inclusion, to avoid any selection bias. Thus, the results of our study should be carefully extrapolated to other population.

## 5. Conclusions

Our study showed that pro-inflammatory cytokine levels of TNF-α and IL-1β were elevated in patients with COPD and could be important biomarkers for COPD and severity of airflow limitation. Dose response was seen in later stages of airflow limitation for both TNF-α and IL-1β. We observed a differential response for elevation of TNF-α and IL-1β among patients with TS-COPD and BMS-COPD. Whether these have any prognostic and therapeutic implications, such as careful selection of patients with specific phenotypes of COPD with elevated TNF-α and IL-1β levels who may benefit from individualized anti-TNF-α and anti-IL-1β treatment, is unclear at present and needs further studies.

## Figures and Tables

**Table 1:** Demographic and clinical details of COPD cases and controls in the study.

Variable	Cases (*n* = 80)	Controls (*n* = 80)	*p* Value
Age in years, mean (SD)	59.38 (8.63)	57.02 (10.68)	0.12616
Body Mass Index	22.42 (4.26)	24.49 (5.02)	0.00559
Smoking Pack years, mean (SD) (males, *n* = 40)	35.55 (9.95)	0.28 (1.26)	0.0000
Biomass exposure index, mean (SD) (females, *n* = 40)	78.9 (13.99)	18.63 (5.63)	0.0000
FEV1 in L, mean (SD)	1.10 (0.43)	2.28 (0.76)	0.0000
FEV1 %(pred), mean (SD)	48.77 (13.57)	98.25 (14.30)	0.0000
FEV1/FVC ratio, mean (SD)	57.6 (7.99)	81.65 (4.8)	0.0000
Six-minute walk distance in metres, mean (SD)	305.53 (55.4)	550.53 (58.4)	0.0000
IL1-β in pg/mL, mean (SD)	27.12 (29.84)	8.30 (1.98)	0.0000
TNF-α in pg/mL, mean (SD)	19.71 (18.89)	15.02 (3.53)	0.03077
SGRQ score, mean (SD)	23.35 (2.92)	-	-
BODE Index, mean (SD)	3.53 (1.92)	-	-
pH, mean (SD)	7.38 (0.03)	-	-
PO2, mean (SD)	82.17 (7.76)	-	-
PCO2, mean (SD)	40.40 (4.12)	-	-

BMI = Body Mass Index; BMEI: Biomass exposure index, BMS: Biomass smoke, BODE: Body mass index (BMI, B), airflow obstruction (O), dyspnea (D), and exercise tolerance (E), COPD: Chronic obstructive pulmonary disease, FVC: Forced Vital Capacity, FEV1: Forced expiratory volume in one second, mMRC: Modified Medical Research Council, SGRQ: St. George’s Respiratory Questionnaire, SMWD: Six-minute walk distance, TS: Tobacco smoke, IL: Interleukin; TNF: Tumor necrosis factor.

**Table 2:** Demographic and clinical details of sub-groups of cases and controls in the study.

Variable	Cases			Control		
	**BMS COPD (*n* = 40, Females)**	**TS COPD (*n* = 40, Males)**	***p* Value**	**Females (*n* = 40)**	**Males (*n* = 40)**	***p* Value**
Age in years, mean (SD)	54.90 (7.22)	60.20 (12.02)	0.00000	53.85 (8. 14)	60.2 (12.03)	0.00707
Body Mass Index	24.04 (4.09)	20.82 (3.84)	0.00038	25.91 (5.11)	23.09 (4.59)	0.01101
FEV1 in L, mean (SD)	0.91 (0.26)	1.29 (0.47)	0.00002	1.70 (0.45)	2.85 (0.53)	0.0000
FEV1 % (pred), mean (SD)	50.80 (13.12)	46.75 (13.89)	0.18	92.1 (10.19)	104.4 (15.27)	0.00006
FEV1/FVC ratio, mean (SD)	61.18 (6.33)	54.03 (8.65)	0.0001	82.63 (4.6)	80.68 (4.9)	0.0632
IL1-β in pg/mL, mean (SD)	19.16 (22.04)	35.08 (34.48)	0.01500	7.76 (1.73)	8.85 (2.09)	0.01304
TNF-α in pg/mL, mean (SD)	15.13 (7.83)	24.28 (24.88)	0.02795	14.54 (3.40)	15.5 (3.64)	0.23226
Six-minute walk distance in metres, mean (SD)	316.3 (42.21)	294.95 (64.86)	0.08748	493.6 (29.4)	589.3 (64.2)	0.0000
SGRQ score, mean (SD)	23.64 (3.04)	23.08 (2.81)	0.38986	-	-	-
BODE Index, mean (SD)	2.93 (1.56)	4.15 (2.08)	0.00386	-	-	-
pH, mean (SD)	7.39 (0.02)	7.39 (0.03)	0.28739	-	-	-
PO2, mean (SD)	82.86 (8.47)	81.5 (7.02)	0.43678	-	-	-
PCO2, mean (SD)	40.89 (2.65)	39.93 (5.20)	0.30516	-	-	-

BMI = Body Mass Index; BMEI: Biomass exposure index, BMS: Biomass smoke, BODE: Body mass index (BMI, B), airflow obstruction (O), dyspnea (D), and exercise tolerance (E), COPD: Chronic obstructive pulmonary disease, FEV1: Forced expiratory volume in one second, mMRC: Modified Medical Research Council, SGRQ: St. George’s Respiratory Questionnaire, SMWD: Six-minute walk distance, TS: Tobacco smoke, IL: Interleukin; TNF: Tumor necrosis factor.

**Table 3:** Linear regression analysis for the cytokines as dependent variable and TS-COPD and BMS-COPD groups with BMI as independent variables.

Groups	Other Variables	TNF-α		IL-1β	
		Coefficient	*p*-Value	Coefficient	*p*-Value
TS-COPD/Controls (M)		8.79	0.0301	26.23	0.000007
TS-COPD/Controls (M)	BMI	8.48	0.0442	27.65	0.000006
BMS-COPD/Controls (F)		0.59	0.67	11.4	0.0017
BMS-COPD/Controls (F)	BMI	0.56	0.69	11.6	0.0018
TS-COPD/BMS-COPD		9.15	0.03	15.9	0.0162
TS-COPD/BMS-COPD	BMI	9.2	0.044	19.03	0.008

BMI = Body Mass Index, BMS: Biomass smoke, COPD: Chronic obstructive pulmonary disease, F: female, M: male, TS: Tobacco smoke, IL: Interleukin; TNF: Tumor necrosis factor.

## Data Availability

The data presented in this study are available on request from the corresponding author.
